# Confounding clinical picture in the diagnosis of left ventricular and valvular masses: a case report

**DOI:** 10.1093/ehjcr/ytad606

**Published:** 2023-11-28

**Authors:** Amrin Kharawala, Yi-Yun Chen, Timothy Christian, Rosy Thachil

**Affiliations:** Department of Medicine, NewYork City Health+Hospitals/Jacobi, Albert Einstein College of Medicine, Bronx, NY, USA; Department of Medicine, NewYork City Health+Hospitals/Jacobi, Albert Einstein College of Medicine, Bronx, NY, USA; Department of Cardiology, NewYork City Health+Hospitals/Jacobi, Albert Einstein College of Medicine, Bronx, NY, USA; Division of Cardiology, NewYork City Health+Hospitals/Elmhurst, Mount Sinai School of Medicine, Queens, NY, USA

**Keywords:** Case Report, Left ventricular mass, Valvular masses, Cardiac MRI, Echocardiogram

## Abstract

**Background:**

Masses in the heart and valves have a broad differential diagnosis including infective and rheumatic causes as well as primary or metastatic tumours. Diagnosis involves delineating the location, shape, and origin of the mass/masses and considering the clinical context. This case outlines the work-up and approach to diagnosing a cardiac mass along with imaging findings of a unique secondary metastatic mass in the left ventricle (LV).

**Case summary:**

A 69-year-old female with past medical history of metastatic lung cancer treated with radiotherapy and breast cancer treated with mastectomy presented with dyspnoea and fever. Due to concern for infective endocarditis, transthoracic echocardiogram (TTE) was performed revealing 2 cm × 0.72 cm finger-like, echo-lucent, mobile mass, appearing to originate from LV lateral wall, protruding into the LV cavity, along with valvular masses on mitral and tricuspid valves. Initial differential diagnosis included benign pathologies, but due to the clinical suspicion of malignancy, cardiac MRI was performed which revealed a broad-based mass with invasion into the LV lateral wall and delayed gadolinium enhancement, suggestive of metastatic tumour. The patient was given Aspirin to prevent embolization and eventually underwent hospice care.

**Discussion:**

Atypical appearing cardiac masses can be seen on TTE. Cardiac magnetic resonance imaging (MRI) should be used for definite diagnosis in cases where clinical features do not match the echocardiographic findings.

**Learning points**

- Not all cardiac masses with an echo-lucent core and cystic appearance on an echocardiogram are benign. Cardiac MRI should be pursued to diagnose cardiac masses with conflicting features.
- A broad range of differentials should be correlated to the patient’s medical history and clinical features in order to reach a definitive diagnosis.

## Introduction

Left ventricular and valvular masses can broadly be divided into benign and malignant categories. The first and critical step of diagnosis of cardiac mass is to differentiate whether the mass is a thrombus, vegetation, or tumour,^1–3^ and multimodality imaging are crucial for this. Transthoracic echocardiogram (TTE) is the initial diagnostic modality of choice for such masses.^4^ Generally, echocardiographic features of mass with echo-lucent core tend to have a benign aetiology, as seen in haemangiomas.^5^ However, further imaging may reveal an alternate but more accurate diagnosis. Transoesophageal echocardiography (TEE) is an appropriate next step to characterize valvular masses by providing a more accurate assessment of the anatomical relationships, size, and shape of the mass.^6^ Cardiac computed tomography (CT) has also been favoured as a diagnostic modality, especially for the evaluation of calcified masses and tumour staging.^7^ Flurodeoxyglucose positron emission tomography (FDG-PET/CT) imaging can also be used to identify metastatic lesions. Endomyocardial biopsy in the diagnosis of cardiac tumour is a class 2a recommendation, especially if the diagnosis cannot be obtained by a non-invasive modality.^8^ The non-invasive diagnostic tool that provides the best tissue resolution is cardiac magnetic resonance imaging (CMR), which reveals the morphology and accurately measures the mass.^9^ The most common modulation of CMR is cine magnetic resonance imaging (MRI) in both long and short-axis views, which provide evaluation of location, morphology, extent, border, and mobility of cardiac masses.^9^ It also further delineates cardiac contractility, valvular dysfunction, pericardial lesions, and extra-cardiac involvement.^9^ We present the case of a 69-year-old female who was found to have metastatic cardiac masses with an atypical appearance on TTE, which was then more definitively diagnosed using CMR.

## Summary figure

**Figure:**
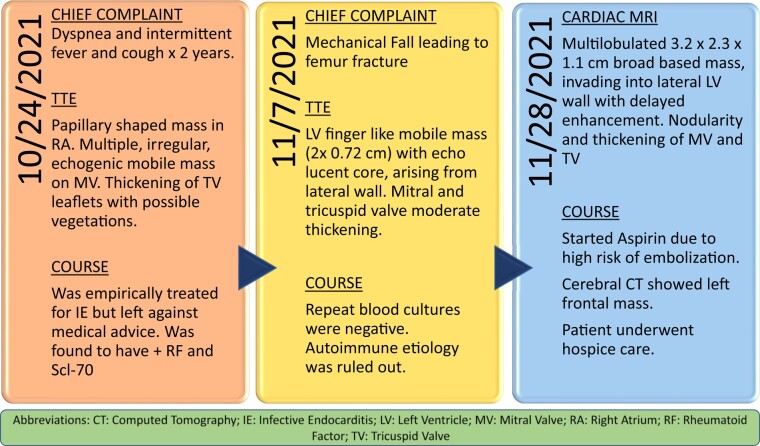


## Case presentation

A 69-year-old African American female initially presented to the emergency department for shortness of breath secondary to chronic obstructive pulmonary disease (COPD) exacerbation. She had extensive past medical history of stage four squamous cell lung cancer with bone metastasis for which she completed radiotherapy treatment, and breast cancer treated with mastectomy. She also experienced chronic intermittent fever, chills, and productive cough with green-yellow sputum since her cancer diagnosis two years ago. She had a 40-pack year smoking history, without history of intravenous drug use. On exam, she was found to be cachexic but in no acute distress, with clear lung exam, and normal heart sounds without murmurs.

**Figure 1:**
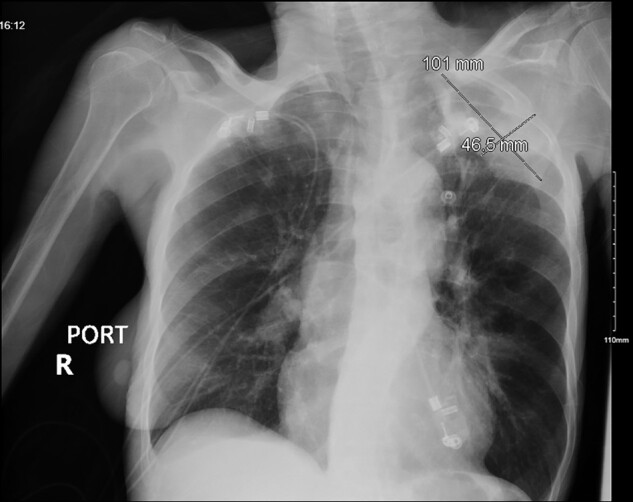
Chest x-ray. 4.6 × 10 cm mass is seen in the left upper lobe.

On laboratory tests, the patient was found to be anaemic at baseline, without leukocytosis, and with venous lactate of 2.4 mmol/L. Blood cultures grew gram-positive cocci in clusters. Inflammatory markers were elevated with C-related peptide (CRP) of 104.8 mg/L (normal: < 3 mg/L) and erythrocyte sedimentation rate (ESR) of 115 mm/hr (normal: < 20 mm/hr). Chest x-ray was significant for a left pleural-based metastatic lung mass, without evidence of consolidation or pleural effusion (*Figure 1*). Electrocardiogram (ECG) showed normal sinus rhythm. Due to concern for endocarditis, TTE was performed, which showed multiple large, irregular, echogenic, highly mobile masses on the tip of the anterior and posterior leaflets of mitral valve that extended to sub-valvular apparatus, involving the papillary muscle. Additionally, a large, papillary-shaped mass was also seen in the inferior right atrial cavity with moderate thickening of the tricuspid leaflets, likely representing vegetation. Patient was also worked-up to rule out non-infective causes of endocarditis; and was found to be positive for rheumatoid factor, anti-citrullinated peptide, and anti-scl-70 antibody, but negative for elevated complement levels.

Initial differential diagnosis included non-bacterial thrombotic endocarditis, infective endocarditis (IE), secondary metastasis, cyst (blood or infectious), tumour, abscess, or thrombus. As blood cultures speciated into methicillin-resistant *Staphylococcus hominis*, another set of blood cultures was obtained before administrating antibiotics and the Department of Infectious Disease (ID) was consulted. The first set of cultures was deemed likely to be contaminants as she had no indwelling catheters or devices and did not spike high-grade fever with leukocytosis, which would make bacteraemia with coagulase negative Staphylococcus unlikely. As repeat cultures came back negative, no further antibiotics were given as per ESC 2015 guidelines, which state that in IE, all blood cultures tend to be positive and a single positive with subsequent negative cultures should be interpreted cautiously.^10^

**Figure 2:**
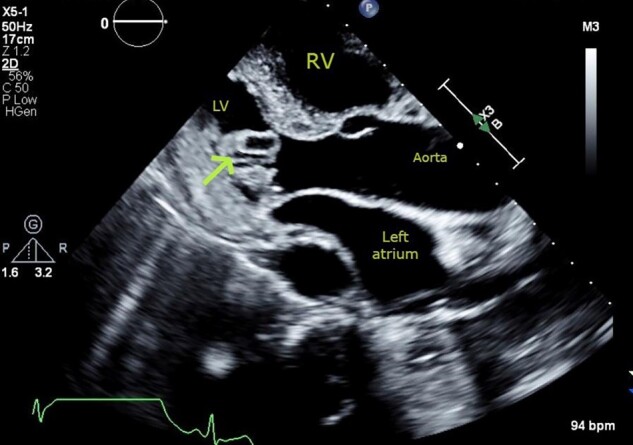
Transthoracic echocardiogram. Parasternal long-axis view showing cystic mass (arrow) with echo-lucent core, protruding into the left ventricular cavity from the basal infero-lateral wall.

**Figure 3:**
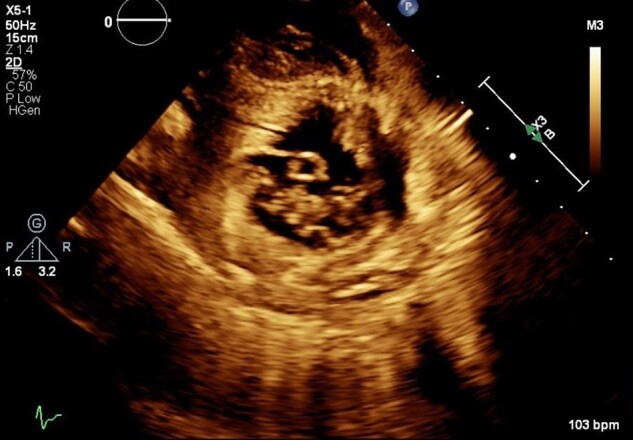
Transthoracic echocardiogram. Basal parasternal short axis at the mitral valve view showing pedunculated finger-like, cystic mass (2×0.72 cm) protruding into the cavity of the left ventricle. Cross sectional view of mass with echo lucent core.

**Figure 4:**
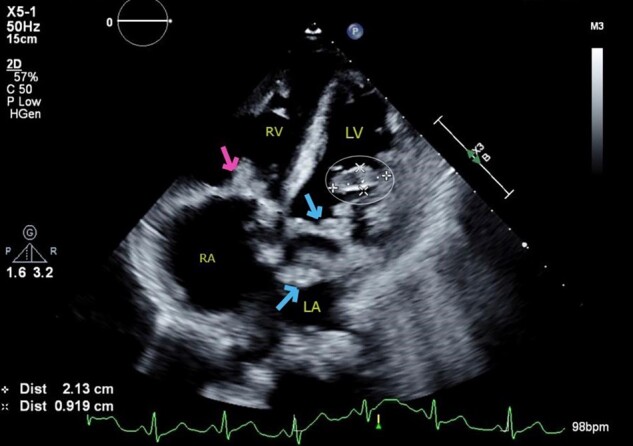
Transthoracic echocardiogram. Apical four chamber view showing cystic mass protruding from the anterolateral wall of the left ventricle into the cavity (cross and circle), along with multiple vegetations (vs. mass) on the mitral valve (arrows) and tricuspid valve (arrow).

**Figure 5:**
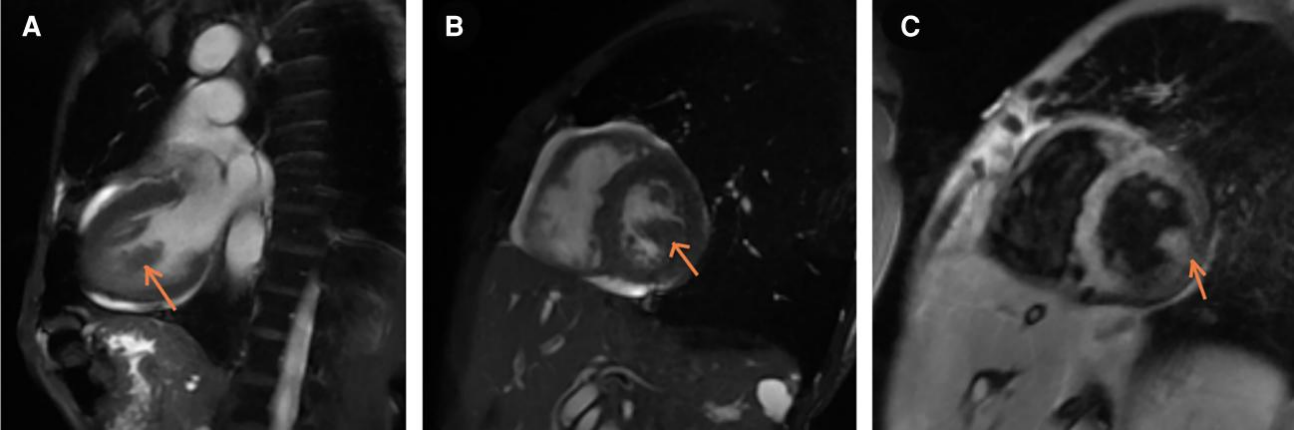
Cardiac MRI. (*A*) Long axis view: Mass from lateral wall, (*B*) Mid Short axis view at the level of papillary muscles: Mass from the lateral wall; same intensity as myocardium itself suggesting a solid mass (as compared to TTE where it had an echo-lucent core as if filled with fluid), (*C*) Gadolinium enhancement: mass enhances in a similar fashion as the lateral wall -> solid mass.

Repeat TTE showed a 2 cm × 0.72 cm finger-like, echo-lucent, mobile mass, originating from LV lateral wall, protruding into the left ventricular (LV) cavity, along with similar valvular masses as before (*Figures 2–4*, Supplementary material online, *Videos S1* and *S2*). The patient did not experience worsening fever, progressive valvular dysfunction, or leukocytosis going against the diagnosis of an infectious aetiology. Rheumatic disease was ruled out as it is unlikely to be associated with vegetation, which is more common in anti-nuclear antibody (ANA) driven diseases with anti-phospholipid syndrome (APS) overlap. Subsequently, she underwent CMR which showed a mobile, multi-lobulated 3.2 × 2.3 × 1.1 cm mass, attached to the lateral wall of the left mid ventricle with delayed enhancement of the mass on perfusion (see *Figure 5*, Supplementary material online, *Video S3*). It was associated with a broad base, suggestive of invasion into the lateral LV wall. The nodularity of mitral valve and thickening of tricuspid valve were also seen. These findings were attributed to cardiac metastasis secondary to lung cancer. She was not deemed a candidate for in-patient chemotherapy. Due to high risk of embolization, patient was started on low-dose aspirin and recommended for follow-up with oncology; however, a month later she was found to have hypercalcemia and a left frontal mass with vasogenic oedema on head CT. She was started on metoprolol 12.5 mg to minimize LV outflow obstruction and anticoagulation was withheld due to brain metastasis. Patient eventually underwent hospice care.

## Discussion

The frequency of primary cardiac tumours is approximately 0.02%.^11^ Metastasis from primary malignancies from lung, breast, ovary, kidney, leukaemia, lymphoma, and malignant melanoma are the most common causes.^3^ Incidence of secondary malignancies is 20 to 40 times higher than that of primary cardiac tumour.^3^ Therefore, TTE should be performed in all patients with a history of malignancy and cardiac symptoms. It is generally uncommon to find cardiac metastasis on valves due to constant motion of the cusps and absence of vessels in the valvular stroma, making this presentation unique.^3^

In our case, TTE initially revealed a cystic mass in the LV, along with multiple valvular masses. As per the ESC 2015 guidelines, we first ruled out IE.^10^ Initial differential diagnosis included benign aetiologies like thrombi, lipoma, and fibroelastoma due to the simultaneous presence on valves. However, given the clinical picture, CMR was pursued and demonstrated that the mass had the same intensity as the myocardium, suggesting a solid mass (in contrast to TTE which showed a seemingly fluid filled echo-lucent core). CMR was also suggestive of invasion of the mass into the free LV lateral wall and showed late gadolinium enhancement, which was attributed to cardiac metastasis secondary to lung cancer. This highlights the importance of pursuing CMR in the setting of a clinical picture contradictory to the TTE findings. Most secondary cardiac tumours are hypointense on T1 weighted image and hyperintense on T2 weighted image with the exception of malignant melanoma.^6^ However, it is important to note that due to low temporal resolution, CMR is not ideal for diagnosing calcification and small masses, especially those related to valves.^6^

Mobile vegetation carries a high risk for systemic embolism and anticoagulation should be continued indefinitely unless there are major bleeding complications.^12^ Low molecular weight heparin (LMWH) and unfractionated heparin have shown superiority in mortality in non-bacterial thrombotic endocarditis in patients with malignancy.^12^ Some studies have suggested that warfarin is less effective than heparin in preventing thromboembolic events in patients with malignancy.^12,13^ In general, the prognosis of cardiac metastasis depends on the primary tumour. However, the metastasis itself confers a stage 4 classification, resulting in poor prognosis.^6^ Treatment of secondary cardiac metastasis is individualized and directed towards the primary tumour. Resection can be performed in select cases where cardiac masses cause haemodynamic compromise.^6^

## Conclusion

In a patient with known metastatic cancer, a finger-like, echo lucent, mobile mass originating in the left ventricle (LV) with invasion into the LV lateral wall, with delayed enhancement on perfusion on CMR, in the absence of clinically significant fever or bacteremia, is most likely to be due to secondary metastasis. Such LV tumours tend to be clinically silent and have a high risk of embolization. Hence, high suspicion is required for diagnosis and prophylactic anticoagulation should be offered to eligible patients.

## Supplementary Material

ytad606_Supplementary_Data

## Data Availability

The data underlying this article are available in the article and in its online Supplementary material.

## Lead author biography

Amrin Kharawala is an Internal Medicine resident at Jacobi Medical Center/Albert Einstein College of Medicine in Bronx, New York, USA. She is interested in pursuing a career in Cardiology and has worked on Cardiology-based research on topics ranging from heart failure, atrial fibrillation, pulmonary embolism, coronary diseases and imaging.

## Notes

**Consent**: The authors confirm that written informed consent for submission and publication of this case report including images and associated text has been obtained from the patient’s next of kin in line with COPE guidance.

**Funding:** No funding was obtained.

**Ethics approval statement:** Not applicable.
